# Adiponectin Ameliorates GMH-Induced Brain Injury by Regulating Microglia M1/M2 Polarization *Via* AdipoR1/APPL1/AMPK/PPARγ Signaling Pathway in Neonatal Rats

**DOI:** 10.3389/fimmu.2022.873382

**Published:** 2022-06-03

**Authors:** Ningbo Xu, Xifeng Li, Jun Weng, Chunhua Wei, Zhenyan He, Desislava Met Doycheva, Cameron Lenahan, Wenhui Tang, Jian Zhou, Yanchao Liu, Qiang Xu, Yahong Liu, Xuying He, Jiping Tang, John H. Zhang, Chuanzhi Duan

**Affiliations:** ^1^Neurosurgery Center, Department of Cerebrovascular Surgery, Engineering Technology Research Center of Education Ministry of China on Diagnosis and Treatment of Cerebrovascular Disease, Zhujiang Hospital, Southern Medical University, Guangzhou, China; ^2^Department of Physiology and Pharmacology, Basic Sciences, School of Medicine, Loma Linda University, Loma Linda, CA, United States; ^3^Department of Hepatobiliary Surgery II, Zhujiang Hospital, Southern Medical University, Guangzhou, China; ^4^Department of Medical Oncology, The Affiliated Tumor Hospital of Zhengzhou University, Zhengzhou, China; ^5^Department of Neurosurgery, The Affiliated Tumor Hospital of Zhengzhou University, Zhengzhou, China; ^6^Department of Biomedical Sciences, Burrell College of Osteopathic Medicine, Las Cruces, NM, United States; ^7^Department of Medical Science, Shunde Polytechnic College, Foshan, China; ^8^Departments of Anesthesiology, Neurosurgery and Neurology, Loma Linda University School of Medicine, Loma Linda, CA, United States

**Keywords:** adiponectin, microglial polarization, GMH, neuroinflammation, hematoma resolution

## Abstract

Adiponectin (APN), a fat-derived plasma hormone, is a classic anti-inflammatory agent. Multiple studies have demonstrated the beneficial role of APN in acute brain injury, but the effect of APN in germinal matrix hemorrhage (GMH) is unclear, and the underlying molecular mechanisms remain largely undefined. In the current study, we used a GMH rat model with rh-APN treatment, and we observed that APN demonstrated a protective effect on neurological function and an inhibitory effect on neuroinflammation after GMH. To further explore the underlying mechanisms of these effects, we found that the expression of Adiponectin receptor 1 (AdipoR1) primarily colocalized with microglia and neurons in the brain. Moreover, AdiopR1, but not AdipoR2, was largely increased in GMH rats. Meanwhile, further investigation showed that APN treatment promoted AdipoR1/APPL1-mediated AMPK phosphorylation, further increased peroxisome proliferator-activated receptor gamma (PPARγ) expression, and induced microglial M2 polarization to reduce the neuroinflammation and enhance hematoma resolution in GMH rats. Importantly, either knockdown of AdipoR1, APPL1, or LKB1, or specific inhibition of AMPK/PPARγ signaling in microglia abrogated the protective effect of APN after GMH in rats. In all, we propose that APN works as a potential therapeutic agent to ameliorate the inflammatory response following GMH by enhancing the M2 polarization of microglia *via* AdipoR1/APPL1/AMPK/PPARγ signaling pathway, ultimately attenuating inflammatory brain injury induced by hemorrhage.

## Introduction

Germinal matrix hemorrhage (GMH) is the most common type of intracranial hemorrhage to occur in preterm neonates and is caused by the rupture of blood vessels in the periventricular subependymal immature region (i.e., germinal matrix) (1). Destruction of the precursor cells within the germinal matrix and post-hemorrhagic hydrocephalus in infants suffering from GMH (2) may lead to the development of significant long-term neurocognitive sequelae, including developmental delay, seizures, and cerebral palsy (3). Unfortunately, no therapies have been shown to be effective in treating neonatal GMH, and the only preventive intervention is perinatal glucocorticoids (4). As the rates of survival in preterm births and neonates have substantially increased in recent decades, GMH-induced neurological deficits are increasingly evolving into a substantial socioeconomic burden. Therefore, safe and effective pharmacologic treatments are desperately needed.

The initial pathological damage of cerebral hemorrhage is the mechanical compression to adjacent tissues caused by hematoma (5). Secondary damage is known to be triggered by blood clots that contribute to neuroinflammation and neurological dysfunction, including post-hemorrhagic hydrocephalus (6), reactive astrocytosis (7), and microgliosis (8). Secondary brain injury has crucial roles in the prognosis of neurological deterioration in hemorrhagic stroke, including intracerebral hemorrhage, subarachnoid hemorrhage, and GMH. As therapeutic developments against primary injury have demonstrated definitive benefits in clinical trials, investigators have instead focused on exploring the mechanisms that underlie post-GMH secondary brain injury in the pursuit of novel targets for treatment.

Increasing evidence suggests that inflammatory reactions play a key role in the pathogenesis of stroke and contribute to secondary brain injury after hemorrhage (9, 10). Microglia/macrophages are the major immune cells of the central nervous system (CNS) and are critical drivers of the neuroinflammatory response after various hemorrhagic brain injuries (11). Upon activation, microglia develop into either classically activated (M1) or alternatively activated (M2) phenotypes, a process termed polarization. M1 microglia are initially present following an insult as they promote an inflammatory response. Conversely, M2 microglia mainly secrete anti-inflammatory cytokines and growth factors, and may facilitate neuronal recovery, hematoma resolution, and vascular remodeling. Additionally, in response to distinct microenvironmental cues, an already fully polarized M1 or M2 sub-population can reverse its phenotype and function (12, 13). Therefore, strategies aiming to promote the phenotypic conversion of microglia from M1 to M2 might provide therapeutic potential for GMH.

Adiponectin (APN) is a plasma hormone predominantly secreted by adipocytes, and is known to exert powerful anti-apoptosis/anti-inflammatory effects in acute and chronic brain injury, including ischemic or hemorrhagic stroke, mainly through the Adiponectin receptor 1 (AdipoR1) and AMP-activated protein kinase (AMPK) pathway (14). We previously found that adiponectin could exert neuroprotective effects by attenuating neuronal apoptosis *via* AdipoR1/AMPK signaling pathway after neonatal rats are subjected to hypoxic ischemic injury (15). Recently, emerging evidence has shown adiponectin signaling pathway has a detrimental role in regulating the immune response *via* microglia from the central nervous system (CNS) (16, 17). Furthermore, AMPK signaling pathway activation could accelerate hematoma resolution and improve neurological outcomes after intracerebral hemorrhage (ICH) (18).

Thus, in this present study, we hypothesize that APN will attenuate neuroinflammation and accelerate hematoma clearance through the AdipoR1/AMPK signaling pathway after GMH *via* regulation of M1/M2 polarization.

## Material and Methods

### Animals

Two hundred and fifty-five Postnatal day (P) 7 Sprague-Dawley neonatal pups (weight = 12-15 g, Harlan, Livermore, CA) of either sex were randomly assigned into either Sham (n=56) or GMH (n=199) groups. Of the 199 rats in the GMH surgery group, no pups were excluded from the study as a result of intraoperative death. All the rat pups were stored in temperature-controlled rooms with *ad libitum* access to drink and chow. The design of experiments, timeline, and number of animals within each group are depicted in **Supplementary Figure S1**.

### Germinal Matrix Hemorrhage (GMH) Induction

GMH induction was conducted in unsexed P7 rats and was accomplished *via* collagenase infusion as previously described (19, 20). Briefly, rat pups weighting between 12g-15g were anesthetized using 3% isoflurane, and anesthesia maintenance was conducted using 1.5% isoflurane intraoperatively. After, a stereotaxic frame was used to fix the pups in place, and the scalp was then sterilized. Next, the bregma was exposed *via* incision, and a burr hole (1 mm) was drilled at 1.6 mm right lateral and 1.5 mm rostral relative to bregma. A 10 µl Hamilton syringe (Hamilton Co, Reno, NV, USA) filled with 0.3 U/µl collagenase solution was fixed to an infusion pump (Harvard Apparatus, Holliston, MA, USA), and the needle was inserted 2.8 mm below the dura with the bevel facing the midline. Next, 0.3 U type VII-S collagenase from *Clostridium histolyticum* (Sigma Aldrich St. Louis, MO, USA) was infused at a rate of 0.3 U/3min, and the needle remained in place for 5 min before being removed at a rate of 1 mm/min to prevent possible leakage. Bone wax was then used to seal the burr hole, and 5-0 silk was then used to suture the incision closed. In the post-operative period, the animals were placed on a 37°C heating pad to recover and awaken from anesthesia. After recovery, the rat pups were returned to the dam. The sham surgery followed the same procedure as described above, but without collagenase infusion. The average duration of surgery for each animal was approximately 30 min.

### Drug Administration

Recombinant human adiponectin protein (rh-APN) (Catalog Number: 450-24, Pepro Tech, NJ, USA) was administered by intranasally at 1 h post-GMH in three different doses (0.05 mg/kg, 0.1 mg/kg, and 0.3 mg/kg) as described previously (15). The rh-APN was administered intranasally once daily for 3 days post-GMH in the short-term experiment or 7 days using the best dosage in the long-term experiment.

### *In Vivo* RNAi

As previously described (15, 19), 2 μl rat derived AdipoR1 siRNA (siRNA ID: s144018, 300 pmol/μl, Life Technologies), AdipoR2 siRNA (siRNA ID: s161926, 300 pmol/μl, Life Technologies), APPL1 siRNA (siRNA ID: RSS306560, 300 pmol/μl, Life Technologies), LKB1 siRNA (siRNA ID: s163340, 300 pmol/μl, Life Technologies), or Scramble siRNA were administered intracerebroventricularly at 24 h pre-GMH induction (1.5 mm posterior, 1.5 mm lateral to the bregma and 1.7 mm deep from the surface).

### Administration of Liposomes

Liposomes (SKU: F10209D, FormuMax Scientific, Sunnyvale, CA, USA) consisting of lipid fluorescent dye, Lipo-DHPE (Fluorescein DHPE), Lipo-Dorsomorphin (Product No. P5199, Dorsomorphin, AMPK inhibitor, Santa Cruz Biotechnology, Dallas, TX, USA) or Lipo-GW9662 (Product No. M6191, GW9662, PPARγ antagonist, Sigma Aldrich, MO, USA) were prepared according to manufacturer’s protocol as has been previously reported (19). The final concentration of liposomal Dorsomorphin (6 µg/µl) and GW9662 (12 µg/µl) was assessed using a microplate reader system (400nm, SpectraMax i3x, Molecular Devices, Thermo Fisher Scientific, Waltham, MA, USA).

### Immunofluorescence Staining

Double Immunofluorescence staining was conducted on the sections of fixed frozen brains as previously described (19, 20). The 8-μm thick slides were rinsed using phosphate-buffered saline (PBS), followed by permeabilization using 0.3% Triton X-100 for 15 min at RT. Next, the slides were incubated with blocking solution (95% PBS, 5% normal donkey serum, and 0.05% Triton X-100) for 2 h. Next, the slides were incubated using the following: mouse anti-Iba1 (ab283319, 1:200, Abcam, USA), rabbit anti-GFAP (ab254082, 1:50, Abcam, USA), rabbit anti-APN (ab181281, 1:50, Abcam, USA), rabbit anti-AdipoR1 (ab70362, 1:100, Abcam, USA), mouse anti-NeuN (ab104224, 1:200, Abcam, USA), rabbit anti-IL-6 (ab179570, 1:100, Abcam, USA), rabbit anti-CD68 (ab283654, 1:100, Abcam, USA), anti-hemoglobin (ab251919, 1:100, Abcam, USA) or rabbit anti-CD206 (ab64693, 1:100, Abcam, USA) at 4°C overnight. After using PBS to wash the slides three times (10 min each time), the sections were incubated using the appropriate fluorescent secondary antibodies (diluted 1:200) (Jackson Immuno Research, West Grove, PA, USA) and counterstained with DAPI (SKU: H-1200-10, Vector Laboratories, Newark, CA, USA). All counts and quantifications were conducted blindly (19, 21).

### Western Blot Analysis

Brain tissues were homogenized with RIPA lysis buffer (sc-24948, Santa Cruz Biotechnology, Dallas, TX, USA) for a duration lasting at least 60 s. The homogenate was centrifuged at 15,000 rpm/min at 4°C for 20 min. The supernatant was then collected, aliquoted, and stored at -80°C. Protein concentrations were determined *via* DC protein assay (Bio-Rad, USA). Western blots were processed according to previously described protocol (15). Next, 30μg of protein from each sample were placed into wells of 10% gels. After which, each was run for 30 min at 80 V, followed by 90 min at 120 V. The proteins were then relocated onto 0.2μm or 0.4μm nitrocellulose membranes at 100 V for 120 min (Bio-Rad, USA). The following primary antibodies were applied to the membranes, which were allowed to incubate overnight at 4°C: anti-APN (anti-rat) (ab181281, 1:1000, Abcam, USA), anti-APN (anti-human) (ab75989, 1:1000, Abcam, USA), anti-AdipoR1 (ab70362, 1:1000, Abcam, USA), anti-AdipoR2 (ab231051, 1:1000, Abcam, USA), anti-APPL1 (sc-271901, 1:1000, Santa Cruz Biotechnology, Dallas, TX, USA), anti-LKB1 (sc-32245, 1:1000, Santa Cruz Biotechnology, Dallas, TX, USA), anti-*p*-AMPK (2535, 1:1000, Cell Signaling Technology, USA), anti-AMPK (5831, 1:1000, Cell Signaling Technology, USA), anti-PPARγ (ab272718, 1:1000, Abcam, USA), anti-CD36 (ab252922, 1:1000, Abcam, USA), anti-CD68 (ab283654, 1:1000, Abcam, USA), anti-CD206 (ab64693, 1:1000, Abcam, USA), anti-IL-6 (ab179570, 1:1000, Abcam, USA), anti-IL-1β (ab254360, 1:1000, Abcam, USA), anti-IL-10 (ab192271, 1:1000; Abcam, USA), and Actin (sc-8432, 1:1000; Santa Cruz Biotechnology, USA). Membranes were washed and incubated in appropriate secondary antibodies (1:2000; Santa Cruz Biotechnology, Dallas, TX, USA) for 2 h at RT. Fiji software (NIH Images, USA) was used to evaluate and assess relative density (21).

### Hemoglobin Assay

Spectrophotometric measurements to assess hemorrhagic volume were conducted as previously described (21). The extracted, frozen forebrain tissues were distributed into individual glass test tubes containing 3 mL of PBS, followed by homogenization for 60 s with the help of Tissue Miser Homogenizer (Thermo Fisher Scientific, Waltham, MA, USA). Next, the erythrocyte membranes were lysed *via* 1* min* of ultrasonication. After which, the products were centrifuged for 30 min, which then allowed the supernatant to be separated from the pellets. Then, Drabkin’s reagent was added (Sigma-Aldrich, USA) into aliquots of supernatant in a ratio of 4:1, which were allowed to react for 15 min. Finally, a spectrophotometer (540; Genesis 10uv; Thermo Fisher Scientific, Waltham, MA, USA) was used to calculate absorbance into a hemorrhagic volume (µl) according to the basis of a standard curve.

### Neurological Tests

Neurobehavioral function was evaluated blindly in a random and unbiased protocol as previously reported (22). Short-term neurological tests, such as righting reflex and negative geotaxis tests, were performed on days 1 to 3 after GMH. Several long-term neurological tests, including water maze, rotarod, and foot-fault, were performed on days 21 to 28 after GMH.

### Righting Reflex Test

The time for each pup to completely rollover from supine to prone was recorded. The maximum recording time was 60 s (3 trials/pup/day). The values from each of the three trials were used to calculate the average.

### Negative Geotaxis Test

Pups were placed head downward onto a 45°C inclined plane, and the time to rotate 180°C was recorded. The maximum duration of each trial was 20 s (3 trials/pup/day). The values from each of the three trials were used to calculate the average.

### Water Maze Test

Morris water maze tests were conducted to assess animal memory and learning capacity as previously described. The apparatus consisted of a metal pool (110 cm in diameter) accompanied by a small platform (11 cm in diameter), in which the pups could climb to escape the water. Swim distance, latency, and velocity were digitally recorded and analyzed using a tracking software (Noldus EthoVision, USA). In the cued test, pups were manually guided to the platform if they had difficulty in locating it, and the platform location was moved every other trial. In the hidden tests, the platform was submerged 1 cm below the water, and the duration of time spent in probe quadrant was recorded.

### Quantitative Real-Time PCR (qRT-PCR) Analysis

To assess gene expression levels, total RNA was isolated from brain tissue using RNeasy kit (Catalog Number: 74106, Qiagen) and cDNA were synthesized using GoScript Reverse Transcriptase (Catalog Number: PRA5000, Promega) following the manufacturer’s instructions as previously described (19). The RNA expression was measured by qRT-PCR with specific primers (**Table 1**) using iQ™5 real-time PCR detection systems (Bio-Rad, USA).

**Table 1:** Primers used in real-time PCR for the target genes.

Gene Name	GenBank Number	Sense (5’-3’)	Anti-sense (5’-3’)
Adiponectin	NM_144744.3	TGTTCTTGGTCCTAAGGGTGAC	CCTACGCTGAATGCTGAGTGA
AdipoR1	NM_207587.2	CAGAAAACCCAGCAGTTGCC	AAAGGAAACACCCACTGCCA
AdipoR2	NM_001037979.1	GGAGTGTTCGTGGGCTTGGG	GCAGCTCCTGTGATATAGAGG
Arginase-1	NM_017134.3	AAGACAGGGCTACTTTCAGGA	CAAGACAAGGTCAACGCCAC
GADPH	NM_001106123.2	GGTTCC GGTTTGTGGAGCAG	TCCGTTTGCATTGCCCAGTA

### Nissl Staining

On day 28 after GMH, we euthanized the rats, harvested the brains, and fixed them in 10% formalin for histology preparation. The brains were sliced into 16μm-18μm thick slices (Leica Microsystems, LM3050S) after being embedded with Optimal Cutting Temperature (OCT, Fisher Scientific, Waltham, MA, USA). Then, the coronal brain slices were stained with 0.5% Cresyl violet, followed by microscopic imaging (Olympus-BX51). Ventricular volume, white matter loss, and cortical thickness were evaluated as reported previously (19).

### Statistical Analysis

All data are presented as mean ± SD. Statistical analysis was performed using SPSS v.24.0 (IBM Corp., USA). Statistical differences were performed using one-way ANOVA with Dunnet’s *post-hoc* test for multiple comparison and two-tailed Student’s t test for two group comparisons. If the difference was *P* ≤ 0.05, it was considered statistically significant.

## Results

### Time Course and Spatial Expression of Endogenous Adiponectin, AdipoR1, and AdipoR2 After GMH

**Figure 1:**
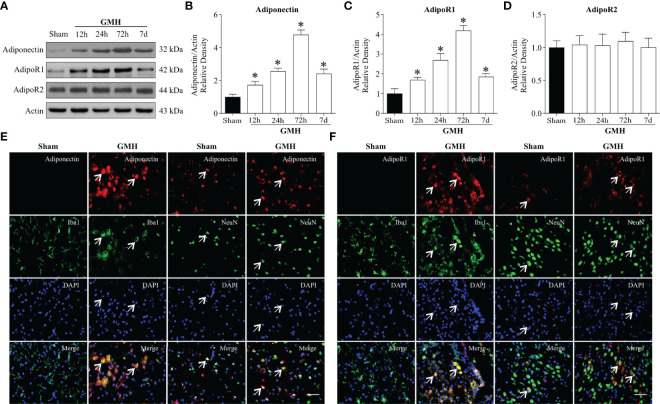
Endogenous Adiponectin and AdipoR1 were upregulated in the brain after GMH. **(A–C)** Western blot data revealed that the endogenous Adiponectin **(A, B)** and AdipoR1 **(A, C)** expression levels significantly increased from 12 h to 3 d and peaked at 3 d post-GMH. **(A, D)** AdipoR2 expression levels were comparable among pups with or without GMH at four time points. Values are expressed as normalized to Actin protein expression. Values are expressed as mean ± SD. ANOVA, Dunnett. *P < 0.05 compared to sham, n = 6 for each group. **(E, F)** Representative images of immunofluorescence staining showing the co-localization of Adiponectin and AdipoR1 (red) with microglia and neurons (Iba1, NeuN, green) in the pups with or without GMH. Adiponectin and AdipoR1 immunoreactivities were greater on microglia and neurons in the periventricular area at 72 h after GMH. Arrows indicated Adiponectin or AdipoR1 colocalized with microglia. Scale bar = 50µm. n = 3 for each group.

Western blot analysis was conducted to assess endogenous Adiponectin, AdipoR1, and AdipoR2 expression at 0 h (Sham), 12 h, 24 h, 72 h, and 7 d in the ipsilateral hemisphere after GMH. The consequent results revealed that the expression of endogenous APN (**Figures 1A, B****)** and AdipoR1 (**Figures 1A, C****)** significantly increased at 12 h, peaked at 72 h, and decreased at 7 d after GMH when compared to Sham group. However, no changes were observed in expression of AdipoR2 after GMH (**Figures 1A, D****)**. Consistently, the patterns of Adiponectin, AdipoR1, and AdipoR2 expression were identified by qRT-PCR analysis (**Supplementary Figures S2B–D**). In addition, double immunofluorescence staining revealed that Adiponectin and AdipoR1 were heavily expressed in microglia, neurons (**Figures 1E, F**) , and astrocytes (**Supplementary Figure S2A**) that were in the proximity surrounding the lateral ventricle of pups with GMH, but were barely expressed in microglia and neurons (**Figures 1E, F****)** found surrounding the periventricular region of pups that had not been subjected to GMH.

### Intranasal Administration of rh-APN Ameliorated Neurological Deficits and Attenuated Inflammation in 72 h After GMH

**Figure 2:**
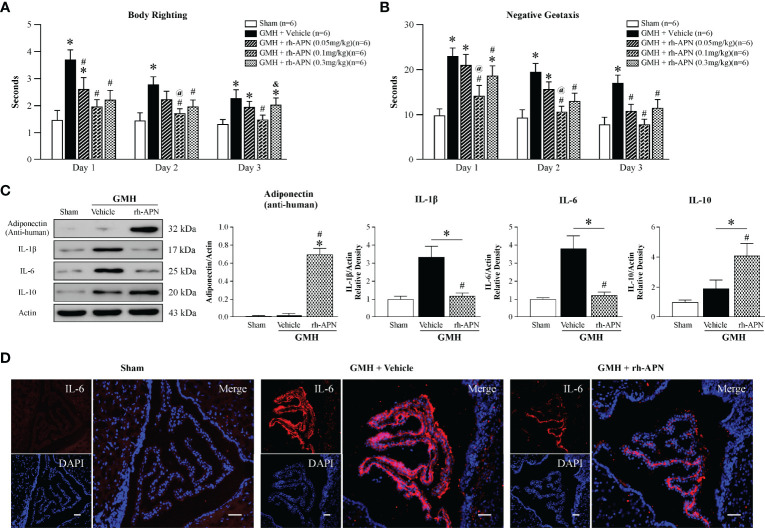
Intranasal administration of Adiponectin improved short-term neurological function at 72 h post-GMH. **(A)** Righting reflex and **(B)** Geotaxis reflex tests revealed that the medium dosage (0.1mg/kg) of rh-APN significantly improved neurological function when compared with the vehicle-treated pups at 1, 2, and 3 d post-GMH. Values are expressed as mean ± SD. ANOVA, Dunnett. n = 6-9 for each group. ^*^P < 0.05 compared to sham, ^#^*P* < 0.05 compared to GMH + Vehicle, ^@^*P* < 0.05 compared to GMH + rh-APN (0.05mg/kg), ^&^*P* < 0.05 compared to GMH + rh-APN (0.1mg/kg). **(C)** Western blot data showed that intranasal administration of rh-APN after GMH could be delivered successfully into brain tissue, and it reduced proinflammatory cytokine levels of IL-1β and IL-6 while increasing expression levels of anti-inflammatory cytokine, IL-10, within the brain. ANOVA, Dunnett. n = 6 for each group. ^*^P < 0.05 compared to sham, ^#^*P* < 0.05 compared to GMH + Vehicle. **(D)** Immunofluorescence staining assay showed IL-6 immunoreactivity in the choroid plexus was dramatically increased in pups that were subjected to GMH when compared with sham, and rh-APN treatment significantly reduced the IL-6 immunoreactivity compared to GMH.

To explore and assess the translational treatment regimen of APN, three doses (0.05mg/kg, 0.1mg/kg, 0.3mg/kg) were administered in a single treatment intranasally 1 h post-GMH. More time was spent flipping to the prone position and rotating to the head upward position in vehicle-treated groups when compared to the Sham group at days 1 and 2 after GMH (**Figures 2A, B****)**. Improved short-term neurological function was observed in all three dosages of rh-APN-treated pups. Interestingly, the medium dose of rh-APN-treated pups demonstrated the best results in both righting reflex (**Figure 2A**) and negative geotaxis tests (**Figure 2B**), which was compared to sham group as early as 2 d post-GMH. Collectively, 0.1mg/kg of rh-APN treatment was used for each of the following experiments.

Anti-human Adiponectin antibody was used to assess exogenous rh-APN levels in the brain after intranasal treatment. As shown in **Figure 2C**, more rh-APN were observed in brain tissue when compared to vehicle treated group. A key feature of stroke involves the secretion of inflammatory cytokines (23). The anti-inflammatory effects of APN have been reported in other studies of intracerebral hemorrhage (ICH) rat models (24). Herein, the protein expression levels of pro-inflammatory (IL-1β and IL-6) and anti-inflammatory cytokines (IL-10) in perihematomal brain tissue were detected. Pro-inflammatory cytokine expression dramatically increased, but anti-inflammatory cytokine expression levels were slightly increased 72 h after GMH (**Figure 2C**). However, with the treatment of rh-APN, the marked increase of pro-inflammatory cytokines (IL-1β and IL-6) were blunted, which was further validated by immunostaining of IL-6 in the choroid plexus (**Figure 2D**). Simultaneously, anti-inflammatory cytokines, IL-10 (**Figure 2C**) and Arginase-1 mRNA expression (**Figure 5H**) by qRT-PCR were significantly promoted.

### Rh-APN Treatment Improved Long-Term Neurological Function at 21 to 28 Days After GMH

**Figure 3:**
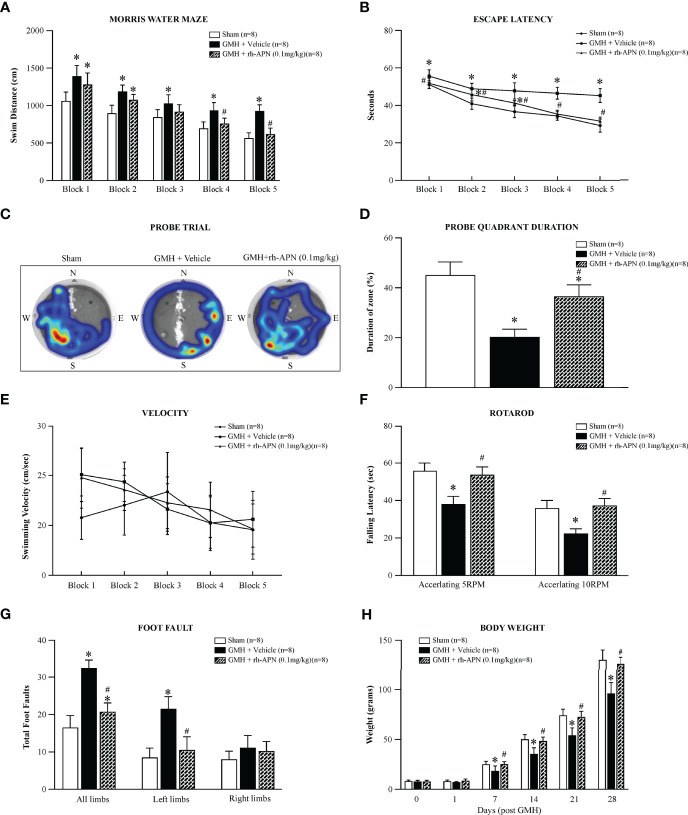
rh-APN administration improved long-term memory and motor function at 21 to 28 days after GMH. Water maze test **(A–E)** showed that rh-APN treatment significantly improved spatial learning and memory performance with reduced swim distance to find the platform **(A)**, less time to escape **(B)**, and more time spent in the probe quadrant **(C, D)**. However, no significant difference in swim velocity was found among the three groups **(E)**. rh-APN treatment notably improved motor function of pups assessed by rotarod **(F)** and foot fault **(G)** tests after GMH. **(H)** Histograms showing the weight changes of pups with or without rh-APN treatment in 4 weeks post-GMH. Values are expressed as mean ± SD. ANOVA, Dunnett. n = 8 for each group. ^*^P < 0.05 compared to sham, ^#^*P* < 0.05 compared to GMH + Vehicle.

To evaluate and explore rh-APN treatment effects on long-term GMH-induced neurological impairments, water maze, foot-fault, and rotarod tests were conducted 4 weeks post-GMH to evaluate and assess neurological function. In the Morris Water Maze evaluation, vehicle-treated rats swam significantly longer (**Figure 3A**), spent a longer duration seeking the platform (**Figure 3B**), and spent less time within the defined quadrant zone (**Figures 3C, D****)** when compared to the sham group. Meanwhile, rh-APN-treated rats had a significantly greater performance than vehicle-treated rats as demonstrated by shorter travel distance, decreased time to escape, and more time spent in the target quadrant zone (**Figures 3A–D**). However, there was no significant difference in swimming velocity among these three groups (**Figure 3E**). Rats in the vehicle groups had a significantly shorter latency to fall at both of 5RPM and 10RPM acceleration compared to the sham group in the rotarod test. Rh-APN treatment significantly reduced the falling latency compared to the vehicle, However, there were no significant differences when compared with sham (**Figure 3F**). Additionally, rh-APN treatment also significantly improved sensorimotor function after GMH in the foot fault test compared to the vehicle group (**Figure 3G**). Regarding the growth profile, there was significantly slowed normal growth from 14 d to 28 d after GMH was observed in vehicle-treated animals compared to sham group, but rh-APN restored normal body weight (**Figure 3H**).

### GMH-Induced Microglial M1 Phenotype Polarization Was Prevented by rh-APN *via* AdipoR1 Signaling Pathway

**Figure 4:**
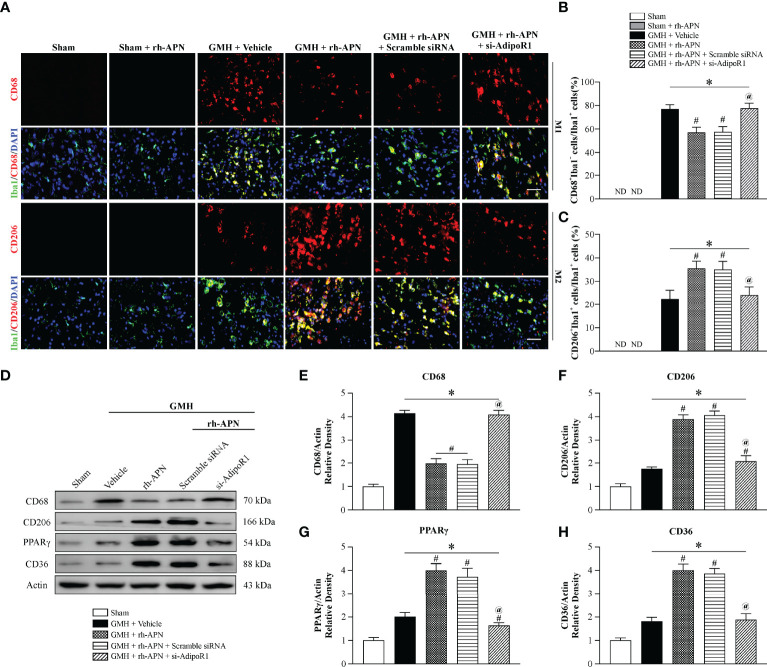
rh-APN treatment promoted M2 microglia polarization at 72 h post GMH. **(A)** Representative images of immunofluorescence staining and quantification **(B, C)** depicting co-localization of CD68 (M1 marker, red) or CD206 (M2 marker, red) and Iba-1 (green). Scale bar = 50μm. **(D)** Representative image of Western blot data showing the expression of CD68, CD206, PPARγ, and CD36. **(E-H)** Western blot quantification showed that the expression of CD68 was markedly increased and CD206, PPARγ, and CD36 expression were slightly increased compared to sham after GMH. **(D–H)** rh-APN significantly decreased CD68 expression, while increased the expression of CD206, PPARγ, and CD36. **(D–H)** However, these effects were reversed by knockdown of AdipoR1 with AdipoR1 siRNA. Values are expressed as mean ± SD. ANOVA, Dunnett. n = 6 for each group. ^*^P < 0.05 compared to sham, ^#^*P* < 0.05 compared to GMH + Vehicle, ^@^*P* < 0.05 compared to GMH + rh-APN or GMH + rh-APN + Scramble siRNA.

There is a beneficial role of alternative polarization of microglia in ameliorating brain hemorrhage-induced inflammation, including GMH (25, 26). To explore whether the microglia M1/M2 polarization involved in attenuating inflammation mediated by rh-APN treatment after GMH, the M1 phenotype marker (CD68) and M2 phenotype marker (CD206) were examined by immunostaining assay. Data showed that the percentage of CD68 and Iba1 double-positive cells increased at 72 h after GMH, but significantly decreased following rh-APN administration (**Figures 4A, B****)**. Simultaneously, rh-APN treatment significantly increased the percentage of CD206 and Iba1 double-positive cells after GMH when compared with the vehicle group (**Figures 4A, C****)**. Importantly, the ratio of M1-like (CD68^+^Iba1^+^) cells/M2-like (CD206^+^Iba1^+^) cells decreased with administration of rh-APN (**Supplementary Figure S3E**). However, the rh-APN-mediated effects in M1/M2 polarization, as described above, were reversed by knockdown of AdipoR1 with si-AdipoR1 (**Figures 4A–C**). In sham animals, microglia remained in their ramified state with or without rh-APN treatment (**Figures 4A–C**).

Consistent with the immunofluorescence staining results, Western blot analysis showed that the expression of M1 marker, CD68, was significantly increased and M2 marker D206 expression was slightly increased at 72 h post-GMH. However, administration of rh-APN significantly decreased CD68 expression and promoted CD206 expression, and the effects were significantly reversed by si-AdipoR1 (**Figures 4D–F**), indicating that rh-APN promoted M2 polarization while inhibiting M1 polarization *via* AdipoR1.

### rh-APN Treatment Promoted Accumulation of Alternatively Activated M2 Microglia in the Periventricular Area After GMH

**Figure 5:**
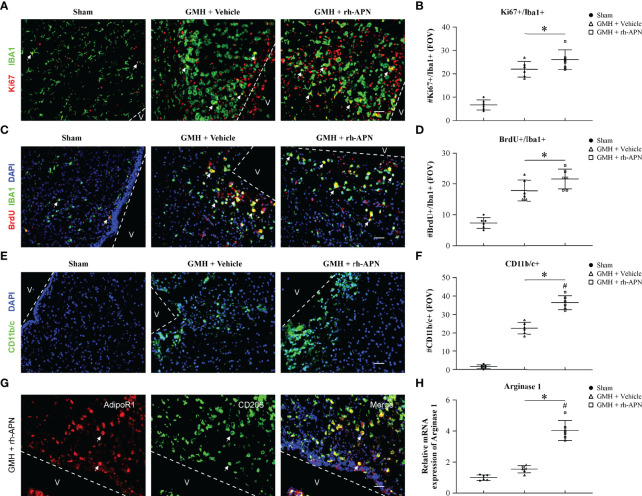
rh-APN promoted M2 microglia accumulation in the periventricular region at 72 h after GMH. **(A, C)** Representative images of immunofluorescence staining and quantification **(B, D)** depicted accumulation and proliferation (Ki67+/Brdu+, red) of microglia (Iba+, green) in the periventricular region. **(E, F)** Representative images of immunofluorescence staining and quantification showing activated microglia/macrophages (CD11b/c+, green) in the periventricular region. Scale bar = 50μm. Dots in **(A-G)** represent data from individual pups. FOV=2.3 × 10^6^ μm^3^. Values are expressed as mean ± SD. ANOVA, Dunnett. n = 6 for each group. ^*^P < 0.05 compared to sham, ^#^*P* < 0.05 compared to GMH + Vehicle. **(G)** Representative images of immunofluorescence staining showing co-localization of AdipoR1 (red) and M2 microglia (CD206+, green) in the periventricular regions after GMH. Scale bar = 50μm. n = 3 for each group. Arrows indicate AdipoR1 colocalized with M2 microglia. **(H)** qRT-PCR assay showed that rh-APN significantly increased the mRNA expression of Arginase-1 at 72 h post-GMH. Values are expressed as mean ± SD. ANOVA, Dunnett. n = 6 for each group. ^*^P < 0.05 compared to sham, ^#^*P* < 0.05 compared to GMH + Vehicle.

Microglia go through various morphological, phenotypic, and functional alterations after GMH induction, which consist of an increase population size *via* proliferation, a transformation from ramified to amoeboid morphology, and chemokine secretion (27). To explore whether treatment with rh-APN affected the post-GMH microglial response, pan microglia marker (Iba1), and activated microglia/macrophage marker (CD11b/c+) were detected in the periventricular area. Iba1 positive-stained microglia in both vehicle and rh-APN-treated groups showed significant morphological changes when compared to sham group at 72 h after GMH (**Figure 5A**).

**Figure 6:**
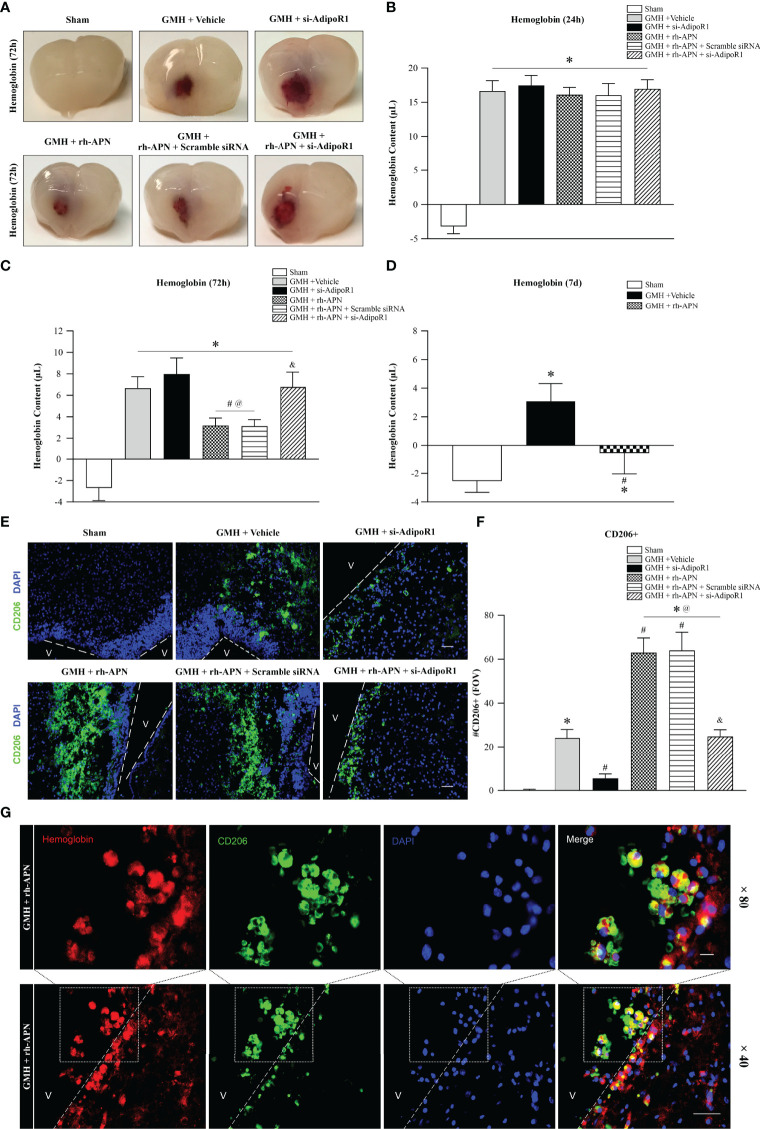
rh-APN promoted hematoma clearance by increasing M2 microglia in the periventricular area after GMH. **(A)** Representative images of hematoma in the periventricular area of brain at 72 h after GMH. **(B–D)** Hemoglobin assays were conducted at **(B)** 24 h, **(C)** 72 h, and **(D)** 7 d. **(E)** Representative images of immunofluorescence staining and **(F)** quantification showing the accumulation of M2 microglia in the periventricular regions after GMH. Scale bar = 50μm. n = 6 for each group. Values are expressed as mean ± SD. ANOVA, Dunnett. n = 6 for each group. ^*^P < 0.05 compared to sham, ^#^*P* < 0.05 compared to GMH + Vehicle, ^@^*P* < 0.05 compared to GMH + si-AdipoR1, ^&^*P* < 0.05 compared to GMH + rh-APN or GMH + rh-APN + Scramble siRNA. **(G)** Representative images of immunofluorescence staining showing co-localization of CD206 with hemoglobin in periventricular area after GMH with rh-APN treatment. Scale bar = 50μm, Upper panel; Scale bar = 50μm, lower panel.

Because microglial proliferation exerts post-hemorrhage protective effects, Ki67^+^/Iba1^+^ microglia, as well as BrdU^+^/Iba1^+^ microglia were evaluated in the periventricular area at 72 h post-GMH. As shown in **Figures 5A–D**, the number of Ki67^+^/Iba1^+^ and BrdU^+^/Iba1^+^ microglia were significantly increased in rh-APN-and vehicle-treated pups compared to sham. However, there is no significant difference between rh-APN and vehicle groups with a slight increase (**Figures 5B, D****)**. Interestingly, the number of activated microglia (CD11b/c^+^) was significantly increased in the periventricular area in rh-APN group when compared with the vehicle group (**Figures 5E, F****)**. Importantly, immunofluorescence staining showed an increased accumulation of AdipoR1+/CD206+ cells (**Figure 5G**) and CD206+ cells (**Figures 6E, F****)** in the periventricular area with rh-APN treatment, indicating that the M2 microglia may account for the large increase in activated microglia.

### rh-APN Administration Enhanced Hematoma Resolution and Reduced Ventricular Dilation by Increasing M2 Microglia in the Periventricular Region After GMH

Peroxisome proliferator-activated receptor gamma (PPARγ) is a pivotal transcription factor and has a key role in the upregulation of CD36 expression (28). Multiple studies have shown that PPARγ is a key participant in promoting polarization of microglia to the M2 phenotype in various experimental stroke models (29, 30). Hence, expression of PPARγ and CD36 expression were evaluated *via* Western blot analysis. The results revealed that exogenous rh-APN administration further augmented PPARγ and CD36 expression at 72 h post-GMH (**Figures 4G, H****)**.

Since a previous study demonstrated PPARγ-induced upregulation of CD36 could enhance hematoma resolution and ameliorate secondary brain injury after GMH (21), hemoglobin assay time-course experiments were performed at 24 h, 72 h, and 7 d post-GMH to further explored the effects of rh-APN treatment on hematoma clearances. At 24 h after GMH, all groups had significantly greater hemoglobin content in the ipsilateral brain compared to sham, but there were no significant differences between GMH groups either with/without treatment of rh-APN or si-AdipoR1 (**Figure 6B**). However, rh-APN treatment had significantly reduced hemoglobin volume when compared with vehicle controls at 72 h after GMH, which had been reversed by AdipoR1 knockdown with si-AdipoR1 RNA (**Figures 6A, C****)**. Additionally, the vehicle- and rh-APN-treated groups had greater hemoglobin content compared to sham at 7 d after GMH, but the rh-APN-treated group had significantly less hemoglobin content compared to the vehicle group (**Figure 6D**).

Given that alternatively activated microglia (M2 phenotype) play a pivotal role in hematoma clearance (5, 21), we evaluated CD206+ microglia in the periventricular region at 72 h post-GMH. There were no CD206+ microglia observed in the sham group, but vehicle-treated pups demonstrated a subtle increase in the number of CD206+ microglia. Importantly, with the knockdown of AdipoR1 by si-AdipoR1 RNA, we observed fewer CD206-positive microglia in si-AdipoR1 group when compared to vehicle groups (**Figures 6E, F****)**. By contrast, treatment of rh-APN significantly increased CD206+ microglia compared to vehicle, whereas this increase was abrogated by knockdown of AdipoR1 (**Figures 6E, F****)**.

Furthermore, double immunostaining showed that CD206 was co-located with hemoglobin (marker of hematoma) in the periventricular area in pups after GMH with rh-APN treatment, indicating rh-APN promoted hematoma resolution by CD206+ microglia mediated phagocytosis (**Figure 6G**).

Additionally, to evaluate whether rh-APN alleviated the severity of ventriculomegaly, the ventricular volume was assessed at 28 d post-GMH. As shown in **Supplementary Figure S3A**, significant ventricular dilation occurred in the vehicle group when compared with sham. However, rh-APN treatment significantly reduced the ventricular volume when compared with the vehicle-treated pups (**Supplementary Figures S3A, B****)**. White matter loss was markedly increased in the vehicle group, but was significantly restored with administration of rh-APN (**Supplementary Figures S3A, D****)**. Concurrently, the cortical thickness was markedly decreased in the vehicle-treated pups, whereas rh-APN treatment significantly reduced the cortical loss (**Supplementary Figures S3A, D****)**.

### rh-APN Attenuates Neuroinflammation and Promotes Hematoma Resolution *via* AdipoR1/APPL1/LKB1/AMPK Signaling Pathway at 72 h after GMH

**Figure 7:**
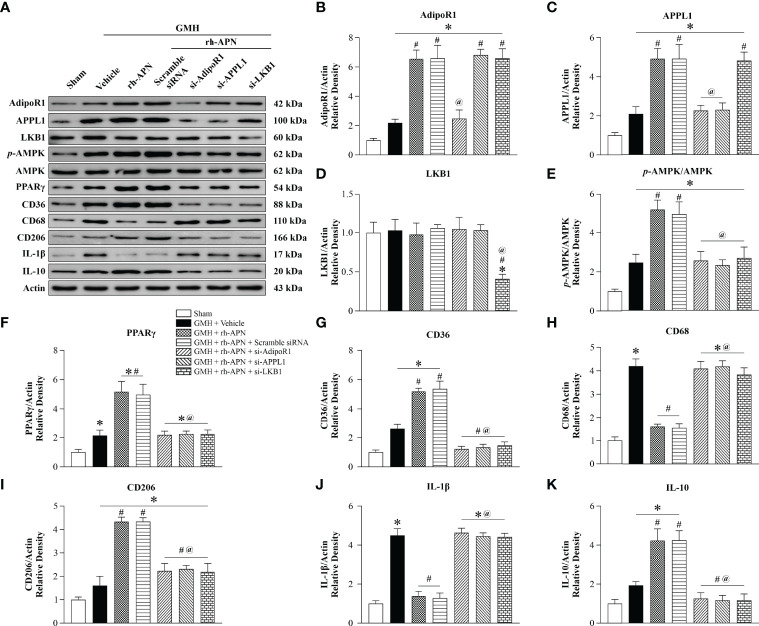
AdipoR1/APPL1/LKB1/AMPK signaling is a potential pathway for rh-APN afforded anti-inflammation in GMH pups. **(A)** Representative images of Western blot data showing the expression of AdipoR1, APPL1, LKB1, p-AMPK, PPARγ, CD36, CD68, CD206, IL-1β, and IL-10 either with rh-APN treatment alone, rh-APN + si-AdipoR1, rh-APN + si-APPL1, or rh-APN + si-LKB1. **(B)** Western blot analysis of AdipoR1 showed that AdipoR1 levels increased in rh-APN treatment group and decreased in the si-AdipoR1 group, while AdipoR1 expression does not change in si-APPL1 or si-LKB1 pups with rh-APN treatment. **(C)** Western blot analysis of APPL1 showed that APPL1 expression was increased in the rh-APN-treated group and decreased in si-AdipoR1 and si-APPL1 groups. However, there were no significant changes of APPL1 levels after knockdown of LKB1 with si-LKB1. **(D)** Western blot analysis of LKB1 showed that LKB1 levels decreased in si-LKB1 treated group. **(E)** Western blot analysis of p-AMPK to AMPK ratio showed that p-AMPK increased in the rh-APN-treated group and decreased in si-AdipoR1, si-APPL1, and si-LKB1 groups. **(F, G, I, K)** Western blot data showed that **(F)** PPARγ, **(G)** CD36, **(I)** CD206, and **(K)** IL-10 expression increased in rh-APN treated group and decreased in si-AdipoR1, si-APPL1, and si-LKB1 groups. **(H, J)** Western blot data showed that **(H)** CD68 and **(J)** IL-1β expression were significantly decreased with rh-APN treatment while si-AdipoR1, si-APPL1, and si-LKB1 reversed the inhibitory effects of rh-APN. Values are expressed as mean ± SD. ANOVA, Dunnett. n = 6 for each group. ^*^P < 0.05 compared to sham, ^#^*P* < 0.05 compared to GMH + Vehicle, ^@^*P* < 0.05 compared to GMH + rh-APN or GMH + rh-APN + Scramble siRNA.

To evaluate and assess whether AdipoR1/APPL1/LKB1/AMPK signaling protect neonatal brain against hemorrhage after GMH, the specific AdipoR1 siRNA, APPL1 siRNA, LKB1 siRNA, and their negative control (scramble siRNA) were intracerebroventricularly injected 24 h before GMH induction. Western blot analysis revealed that AdipoR1, APPL1, p-AMPK, PPARγ, CD36, CD206, and IL-10 expression were each significantly increased in the vehicle group when compared with sham group (**Figures 7A–C, E–H, K**). Administration of rh-APN further upregulated their expression compared to the vehicle-treated group. In contrast, the M1 marker CD68 (**Figures 7A, I****)** and proinflammatory cytokine IL-1β (**Figures 7A, G****)** expression was dramatically increased, whereas rh-APN treatment decreased their expression when compared with the vehicle group. Knockdown of AdipoR1 with AdipoR1 siRNA (si-AdipoR1) significantly decreased AdipoR1 expression and abolished the effects of rh-APN (**Figures 7A–K**). As shown in **Figure 7A**, silencing of AdipoR1 expression was associated with decreased APPL1, AMPK phosphorylation, PPARγ, CD36, CD206, and IL-10 expression, but was associated with increased CD68 and IL-1β expression after GMH with rh-APN treatment. Furthermore, knockdown of APPL1 and LKB1 expression showed similar trends as knockdown of AdipoR1 was associated with decreased AMPK phosphorylation and PPARγ, CD36, CD206, and IL-10 expression (**Figures 7A, F–H, K**), but with increased CD68 and IL-1β expression (**Figures 7A, I, J****)**.

### Selective Inhibition of AMPK/PPARγ Signaling in Activated Microglia Cells Abolished the Effect of rh-APN on Inhibition of Neuroinflammation and Promotion of Hematoma Resolution

**Figure 8:**
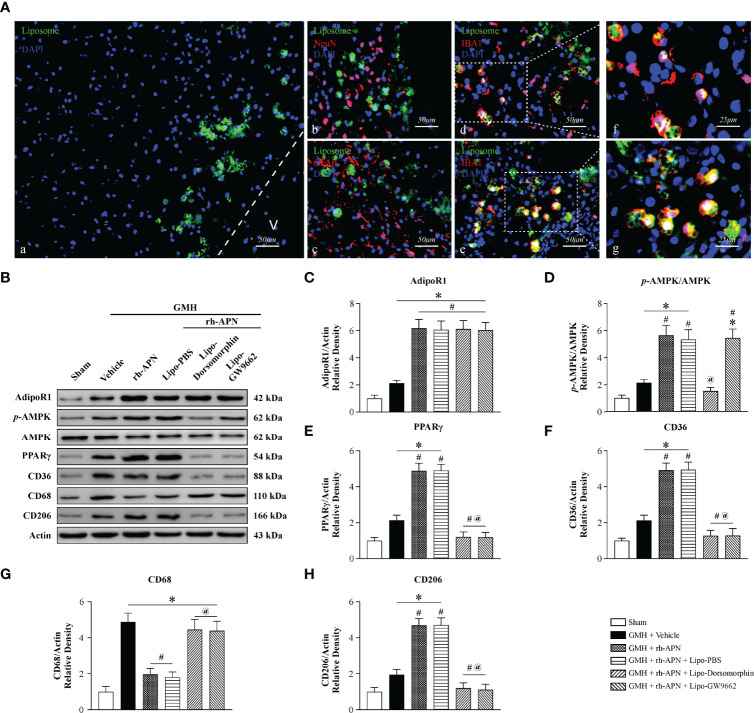
Selective inhibition of AMPK/PPARγ signaling in activated microglia cells abolished the effect of rh-APN on inhibiting neuroinflammation and promoting hematoma resolution 72 hours after GMH **(A)** Representative images of immunofluorescence staining showing fluorescent dye-labeled liposomes **(A**, green**)** were swallowed almost entirely in microglia **D, E** (Iba1^+^, red) rather than in Neurons **(B)** (NeuN^+^, red) and Astrocytes **(C)** (GFAP^+^, red) at 72 h after GMH. **(B)** Representative images of Western blot data showing the expression of p-AMPK, PPARγ and CD36, as well as CD68 and CD206 either with rh-APN treatment alone, rh-APN + Lipo-Dorsomorphin or rh-APN + Lipo-GW9662. **(C)** No changes observed in the expression of AdipoR1 with Lipo-Dorsomorphin and Lipo-GW9662 intervention. **(D)** Western blot analysis of p-AMPK to AMPK ratio showed p-AMPK increased in the rh-APN treatment group and decreased in Lipo-Dorsomorphin group. **(E, F, H)** Western blot data showed **(E)** PPARγ, **(F)** CD36 and **(H)** CD206 expression increased with rh-APN treatment but decreased in Lipo-Dorsomorphin and Lipo-GW9662 groups. **(G)** Western blot data showed that rh-APN significantly decreased CD68 expression, whereas Lipo-Dorsomorphin and Lipo-GW9662 reversed the inhibitory effect of rh-APN. (All samples of GMH + rh-APN in Western blot were from the same animals which were euthanized after short-term neurobehavioral tests). Values are expressed as mean ± SD. ANOVA, Dunnett. n = 6 for each group. ^*^P < 0.05 compared to sham, ^#^*P* < 0.05 compared to GMH + Vehicle, ^@^*P* < 0.05 compared to GMH + rh-APN or GMH + rh-APN + Lipo-PBS.

To specifically assess whether microglia AMPK/PPARγ signaling mediated a protective effect of rh-APN post-GMH, we administered a specific AMPK inhibitor and PPARγ antagonist into microglia cells intracerebroventricularly with Lipo-Dorsomorphin (1μg/g, 2μl) and Lipo-GW9662 (2μg/g, 2μl) 24 h before GMH was induced as described in our previous study (19). Green fluorescence-labeled liposomes were mainly found in activated IBA+ microglia at 72 h post GMH, whereas no labeled liposomes were observed in Neurons (Red, NeuN+) and Astrocytes (Red, GFAP+) (**Figure 8A**).

The addition of Lipo-Dorsomorphin and Lipo-GW9662 to rh-APN had no effect on AdipoR1 expression (**Figures 8B, C****)**. Phosphorylation of AMPK (**Figures 8B, D****)** and PPARγ (**Figures 8B, E****)** expression were significantly reduced in Lipo-Dorsomorphin-treated pups when compared to rh-APN with or without Lipo-PBS-treated pups. In addition, Lipo-GW9662 inhibited PPARγ expression, but exerted no effect on AMPK phosphorylation (**Figures 8B, E, F****)**, indicating that PPARγ is a downstream kinase of AMPK after GMH. Administration of the two liposome-encapsulated inhibitors significantly increased CD68 expression but decreased CD36 and CD206 expression when compared with rh-APN or rh-APN + Lipo-PBS treated pups (**Figures 8B, F–H**). Collectively, AMPK/PPAR**γ** inhibition in activated microglial cells restored post-GMH inflammation, indicating a key role of microglial AMPK/PPARγ signaling to protect the neonatal brain from damage induced *via* hemorrhage.

## Discussion

Germinal matrix hemorrhage (GMH) is the most commonly occurring neurological disorder associated with premature newborns, with an incidence of 3.5 per 1000 live births (31), and is defined as blood vessel rupture of subependymal immature near the ganglionic eminence (32). Herein, we investigated the protective effects of APN on post-GMH brain injury and shed light on mechanisms that could be involved. We found that rh-APN could improve short- and long-term neurobehavioral outcomes by attenuating GMH-induced inflammation. Mechanistically, rh-APN decreased proinflammatory cytokines and increased anti-inflammatory cytokine secretion by preventing GMH-induced microglial polarization to the M1 phenotype while promoting the acquisition of the M2 phenotype. Furthermore, rh-APN enhanced hematoma resolution by promoting accumulation of M2 microglia with enhanced phagocytic ability in the periventricular regions. Finally, Western blot data revealed that knockdown of AdipoR1, APPL1, and LKB1, as well as selectively inhibited AMPK phosphorylation and PPARγ in activated microglia reversed APN’s effects. Taken together, in the present study, we found that APN facilitated the conversion of the M1 to the M2 microglial phenotype after GMH. AdipoR1-mediated activation of the APPL1/LKB1/AMPK/PPARγ pathway had a critical role in the M1 to M2 transformation, as well as in the process of hematoma resolution.

Growing evidence suggests that marked inflammation has crucial roles in GMH-induced secondary brain injury (33, 34). Therefore, we evaluated inflammatory-related cytokines after GMH. Hemorrhagic brain injury in the setting of GMH induces a substantial increase in the production of pro-inflammatory cytokines (IL-1β and IL-6) within the brain, which may consequently progress to hydrocephalus. The post-GMH inflammatory response aggravated GMH-induced hydrocephalus, consequently incurring damage to tissues, disruption of the blood-brain barrier (BBB), and massive brain cell death. Following hemorrhagic stroke, the damaged neurons and astrocytes release various pro-inflammatory molecules, resulting in the activation of microglia to further increase the release of inflammatory cytokines (35, 36). Microglia are capable of acquiring various levels of activation, which reflects the features responsible for their contribution to vital components of recovery after GMH, such as neuroinflammation, tissue repair, or immunomodulation. Classically activated (M1, CD68+) microglia reportedly release destructive pro-inflammatory mediators, whereas the alternatively activated (M2, CD206+) microglia participate in tissue debris clearance *via* phagocytosis, as well as participate in the release of several protective and trophic factors. M1 microglia polarization mainly act in the acute phase after GMH, resulting in substantial pro-inflammatory cytokine release (26). Consistently, microglia primarily polarized to the M1 phenotype with few M2 phenotype microglia at 72 h after GMH in our study.

Microglia/macrophages are characterized by remarkable plasticity and versatility, such as being able to switch from one phenotype to another. After including ICH in mice using collagenase, the switch from the M1 to the M2 microglial phenotype was observed on days 1 to 3 post-ICH (37). Stimulation of cannabinoid receptor 2 (CB2R) suppressed neuroinflammation *via* regulation of M1/M2 polarization *via* cAMP/PKA signaling pathway in an experimental rat model of GMH (26). Recently, we reported that GW9508-mediated GPR40 activation ameliorates neuroinflammation and improves neurological function through PAK4/CREB/KDM6B signaling pathway after GMH (20). Our data from this study showed that intranasal administration of rh-APN promoted M2 microglia polarization with increased anti-inflammatory cytokine (IL-10 and Arginase-1) release.

Moreover, we investigated adiponectin’s effects on sham animals, which revealed that adiponectin did not influence the polarization of microglia in the absence of GMH. Combined with IHC staining, it was shown that rh-APN did not affect activation of microglia in sham animals. It was demonstrated that rh-APN drives microglial M2 polarization on the basis of an activated state. Furthermore, it was revealed that knockdown of AdipoR1 reversed the effect of rh-APN and increased expression of AdipoR1 on alternatively activating (M2, CD206-positive) microglia after rh-APN treatment. In conjunction, it is very likely that the necessary transition from the M1-activated state to the M2 phenotype using rh-APN relies on upregulation of AdipoR1 expression.

Proliferation and accumulation of microglia have detrimental roles pertaining to GMH pathology in the immature preterm brain (38). Tang et al. reported that CB2R activation inhibited thrombin-induced microglial proliferation, as well as the inflammatory response. We found increased microglia proliferation following GMH and rh-APN treatment had no effect on the proliferation of GMH. However, the increased accumulation of activated microglia or macrophages (CD11b/c+) in the periventricular region were observed post-GMH with rh-APN treatment. Meanwhile, administration of rh-APN significantly increased M2 (CD206^+^) microglia, which indicates that the activated and accumulated microglia in the periventricular region might be predominantly M2 (CD206+) microglia.

Peroxisome proliferator-activated receptor-γ (PPARγ) is a ligand-activated transcription factor that belongs to the superfamily of nuclear hormone receptors. Activation of PPARγ mediated the conversion of microglia phenotype and phagocytic capabilities of peripheral M2 polarized macrophages in various CNS diseases (39, 40), including hemorrhagic stroke (21). Flores et al. revealed that the PPARγ-induced CD36 upregulation in microglia increased M2 microglia polarization and was crucial in enhancing post-GMH hematoma resolution.

PPARγ agonists enhanced myelination, reduced inflammation and hydrocephalus, and promoted neurological recovery in newborns with intraventricular hemorrhage (IVH) (41). More recently, studies have shown that activation of adiponectin receptor with adipoRon could boost PPARγ expression and inhibit pro-inflammatory microglia responses, therefore ameliorating hyperperfused cognitive deficits (40). Accordingly, we demonstrate that APN promote phenotypic change of microglia from “classically” M1 activated to “alternatively activated” M2 states and enhances hematoma clearance, thereby reducing hydrocephalus by activation of PPARγ *via* AdipoR1 signaling. Emerging evidence has shown that the activation of AMPK promoted microglial M2 polarization, thereby inhibiting neuroinflammation after stroke (42–44). Moreover, APN ameliorated intracerebral hemorrhage-induced neuroinflammation through the AdipoR1-AMPK pathway (45). Since APPL1 and LKB1 were involved in APN/AdipoR1-mediated AMPK activation (15), AMPK might participate in APN-induced upregulation of PPARγ. Intraventricular administration of specific siRNA-targeted APPL1 and LKB1 significantly reversed AMPK activation and increased PPARγ expression induced by rh-APN/AdipoR1. In addition, CD36 and IL-10 expression were decreased, while IL-1β and IL-6 expression were increased after knockdown of APPL1 and LKB1 was followed with APN treatment, suggesting that APN alleviated GMH-induced inflammation by upregulation of PPARγ *via* AdipoR1/APPL1/LKB1/AMPK signaling.

Because AdipoR1 expression can be found on various brain cells and the role of microglial AdipoR1/AMPK/PPARγ signaling in neuroinflammation *in vivo* remains unexplored, Lipo-Dorsomorphin (AMPK inhibitor) and Lipo-GW9662 (PPARγ antagonist) was used to selectively inhibit AMPK and PPARγ activation in microglia, as previously described (19). With the inhibition of AMPK activation and decreased PPARγ expression, the CD36 and IL-10 expressions were completely reversed with rh-APN treatment. By contrast, expression of IL-1β and IL-6 were significantly increased, suggesting that rh-APN attenuated neuroinflammation may be primarily mediated through the AMPK/PPARγ pathway in microglia. However, our present study mainly focused on the effects of APN, which were mediated *via* microglial signaling after GMH. The APN effects on neurons or astrocytes will be investigated in future studies.

In the present study, GMH was induced *via* stereotaxic injection of sterile collagenase into the ganglionic eminence of newborn rats (P7). However, there are several potential limitations of this model. First, the rapid progression of developmental events following the P7 developmental stage in the rat brain differs from the neonatal human brain, which has been reviewed by others (46). Although researchers have considered the P7 rat to be equivalent to the human term neonate for decades (47), there is evidence that P7 may actually represent an earlier gestation (48, 49). Second, the GMH collagenase model may not only produce hemorrhage, but may also induce a significant inflammatory reaction (46). The increased inflammation may account for a proportion of the enlarged ventricular dilation when compared to that of the GHM model using direct blood injection. Third, the hemorrhage in the brain was not proven to initiate from the germinal matrix. Thus, further investigations are needed to demonstrate the process of hematoma formation following infusion of collagenase into the periventricular ganglionic eminence. Fourth, all pups suffered from the procedure without mortality across the study. This is different from human findings following high-grade GMH, which have a higher rate of mortality (30%-50%) in clinical settings (50).However, the neurological consequences following GMH induced by collagenase successfully mimic the ventricular dilation and the motor deficits seen in human preterm infants.

## Conclusions

**Figure 9:**
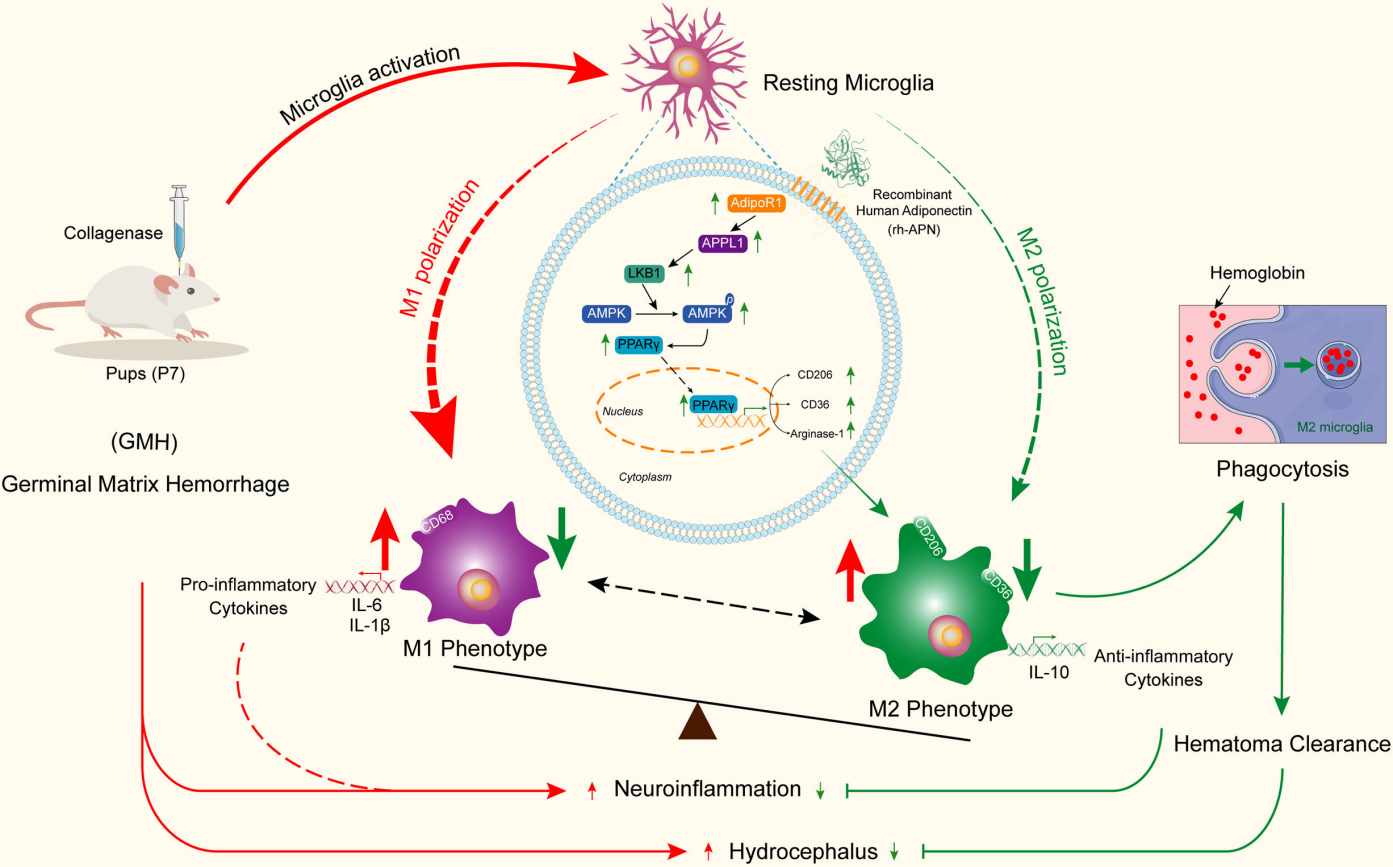
Adiponectin regulates microglia polarization and exerts neuroprotective effects *via* AdipoR1/APPL1/AMPK/PPARγ signaling pathway.

To conclude, this study elucidates the neuroprotective effect of APN in reducing proinflammatory cytokine release and in enhancing hematoma resolution *via* promotion of M2 microglial polarization and accumulation of M2 microglia in the periventricular area, thereby alleviating neurological deficits in an experimental rat model of GMH (**Figure 9**). The neuroprotective effects of APN were associated with the AdipoR1/APPL1/AMPK/PPARγ signaling pathway.

## Author’s Note

This manuscript has been released as a pre-print at ResearchGate [10.21203/rs.3.rs-1280734/v1].

## Data Availability Statement

The original contributions presented in the study are included in the article/**Supplementary Material**. Further inquiries can be directed to the corresponding authors.

## Ethics Statement

The animal study was reviewed and approved by Loma Linda University Institutional Animal Care and Use Committee.

## Author Contributions

NX, XL, DD, JT, JZ, and CD conceived the research idea and experimental design. NX, XL, JW, CW, ZH, WT, CL, JZ, YCL, QX, and YHL performed experiments and analyzed the data. NX, XL, JW, CL, and XH drafted the manuscript. Critical revisions of the manuscript were made by all authors. CD approved the final version of the manuscript on behalf of all the authors.

## Funding

This study received funds from the National Institutes of Health (NS081740 and NS082184), the Medical Scientific Research Foundation of Guangdong Province (A2019377), and the National Natural Science Foundation of China (8203179).

## Conflict of Interest

The authors declare that the research was conducted in the absence of any commercial or financial relationships that could be construed as a potential conflict of interest.

## Publisher’s Note

All claims expressed in this article are solely those of the authors and do not necessarily represent those of their affiliated organizations, or those of the publisher, the editors and the reviewers. Any product that may be evaluated in this article, or claim that may be made by its manufacturer, is not guaranteed or endorsed by the publisher.

## Supplementary Material

Supplementary Figure 1 Timeline of experiment design. GMH, Germinal matrix hemorrhage; WB, Western Blot; rh-APN, recombinant human Adiponectin.

Supplementary Figure 2 Representative images of immunofluorescence staining showing the co-localization of Adiponectin (red) and AdipoR1 (red) with astrocyte (**A**, GFAP, green) in the pups with or without GMH. Immunoreactivities of Adiponectin and AdipoR1 were greater on astrocytes in the periventricular area. Arrows indicate co-localization of Adiponectin or AdipoR1 with astrocytes. Scale bar = 50μm. n = 3 for each group. **(B, C)** qRT-PCR analysis showed mRNA levels of Adiponectin **(B)** and AdipoR1 **(C)**, not AdipoR2 **(D)** increased significantly at 12 h after GMH when data were normalized to GAPDH. Values are expressed as mean ± SD. ANOVA, Dunnett. n = 6 for each group. ^*^*P* < 0.05 compared to sham.

Supplementary Figure 3 **(A–D)** Quantifications of Nissl-stained brain sections showing rh-APN significantly reduced ventricular volume **(A, B)** and the white matter loss **(C)**, while increased **(D)** cortical thickness in pups with GMH. **(E)** The ratio of CD68^+^Iba1^+^ M1-like cells and CD206^+^Iba1^+^ M2-like cells after GMH with rh-APN treatment or rh-APN + Scramble siRNA or rh-APN + si- AdipoR1. Values are expressed as mean ± SD. ANOVA, Dunnett. n = 8 for each group. ^*^*P* < 0.05 compared to sham, ^#^*P* < 0.05 compared to GMH + Vehicle, ^@^*P* < 0.05 compared to GMH + rh-APN or GMH + rh-APN + Scramble siRNA.

Supplementary Figure 4 **(A)** Righting reflex and **(B)** Geotaxis reflex tests showed that intracerebroventricular administration of si-AdipoR1, si-APPL1 and si-LKB1, but not si-AdipoR2, significantly reversed the protective effect of rh-APN when compared with their negative control (Scramble siRNA) group at day 3 after GMH. ^*^*P* < 0.05 compared to sham, ^#^*P* < 0.05 compared to GMH + Vehicle, ^@^*P* < 0.05 compared to GMH + rh-APN or GMH + rh-APN + Scramble siRNA. **(C)** Righting reflex and **(D)** Geotaxis reflex tests showed that Lipo-dorsomorphin and Lipo-GW9662 significantly abolished the protective effect of rh-APN when compared with GMH + rh-APN + Lipo-PBS group at day 3 after GMH. ^*^*P* < 0.05 compared to sham, ^#^*P* < 0.05 compared to GMH + Vehicle, ^@^*P* < 0.05 compared to GMH + rh-APN or GMH + rh-APN + Lipo-PBS. **(E)** Hemoglobin assays were conducted after intracerebroventricular administration of si-APPL1, si-LKB1, Lipo-dorsomorphin, Lipo-GW9662, and their negative control with treatment of rh-APN at 72 hours after GMH. ^*^*P* < 0.05 compared to sham, ^#^*P* < 0.05 compared to GMH + Vehicle, ^@^*P* < 0.05 compared to GMH + rh-APN or GMH + rh-APN + Scramble siRNA. ^&^*P* < 0.05 compared to GMH + rh-APN or GMH + rh-APN + Lipo-PBS. **(F)** The mRNA expression of AdipoR2 with the treatment of rh-APN, rh-APN + si-AdipoR2, or their negative control after GMH by qRT-PCR. ^*^*P* < 0.05 compared to GMH + rh-APN or GMH + rh-APN + Scramble siRNA.
