# Supplementary figures and images for: Adiponectin Ameliorates GMH-Induced Brain Injury by Regulating Microglia M1/M2 Polarization Via AdipoR1/APPL1/AMPK/PPARγ Signaling Pathway in Neonatal Rats
Source: Front Immunol. 2022 Jun 3;13:873382. doi: 10.3389/fimmu.2022.873382 (PMC9203698; doi:10.3389/fimmu.2022.873382)

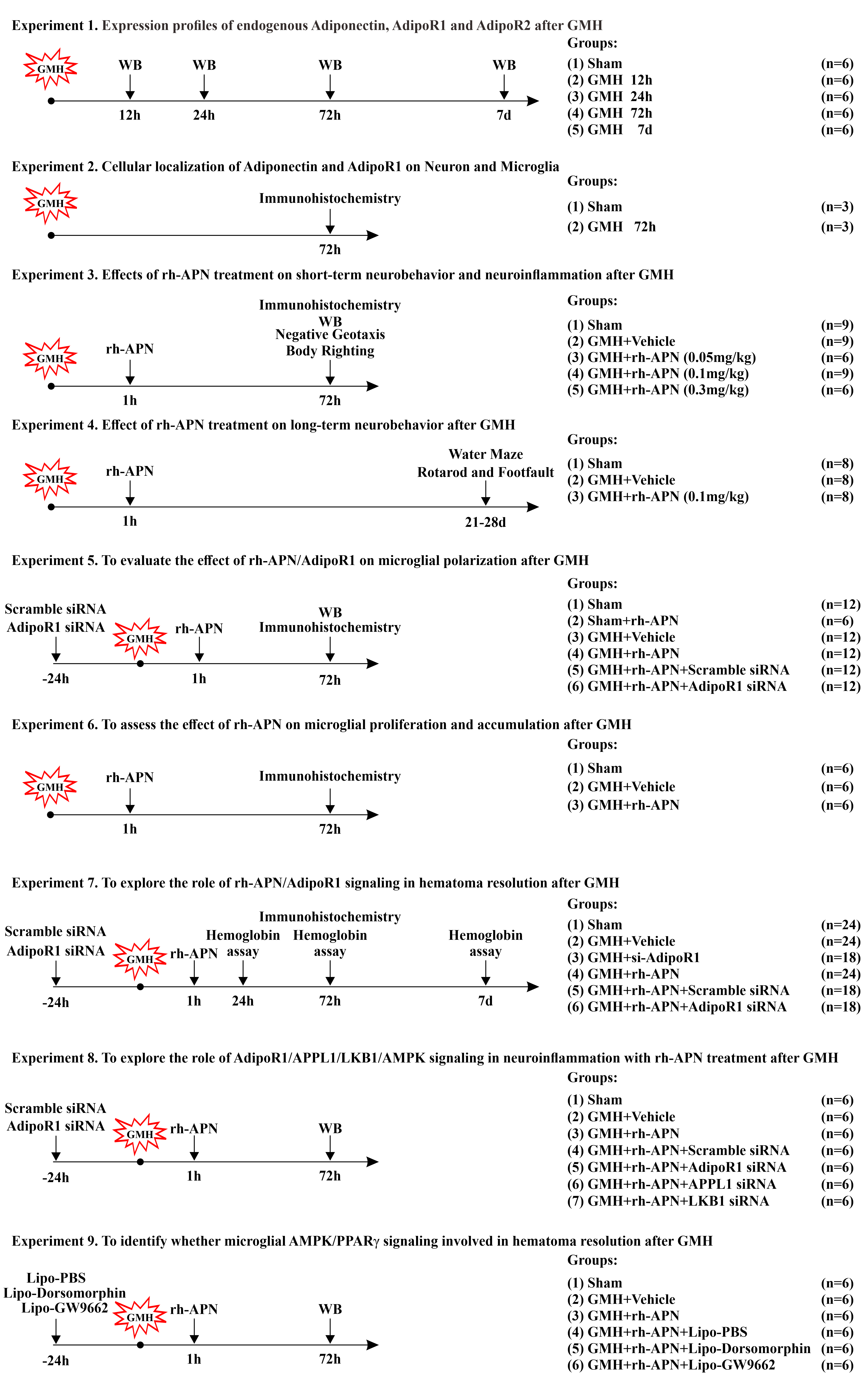

Supplement: Supplementary Figure 1 — Timeline of experiment design. GMH, Germinal matrix hemorrhage; WB, Western Blot; rh-APN, recombinant human Adiponectin. [file Image_1.tif]

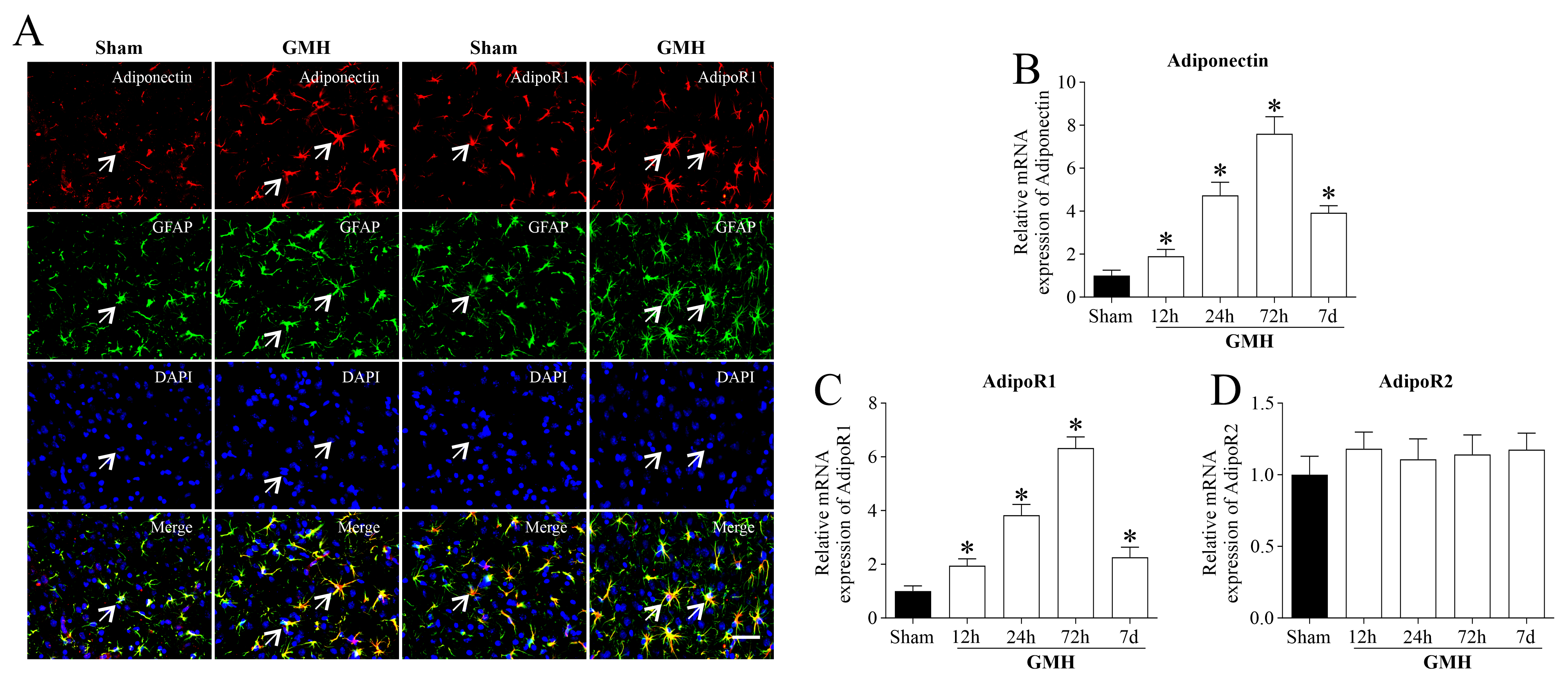

Supplement: Supplementary Figure 2 — Representative images of immunofluorescence staining showing the co-localization of Adiponectin (red) and AdipoR1 (red) with astrocyte (A, GFAP, green) in the pups with or without GMH. Immunoreactivities of Adiponectin and AdipoR1 were greater on astrocytes in the periventricular area. Arrows indicate co-localization of Adiponectin or AdipoR1 with astrocytes. Scale bar = 50μm. n = 3 for each group. (B, C) qRT-PCR analysis showed mRNA levels of Adiponectin (B) and AdipoR1 (C), not AdipoR2 (D) increased significantly at 12 h after GMH when data were normalized to GAPDH. Values are expressed as mean ± SD. ANOVA, Dunnett. n = 6 for each group. *P < 0.05 compared to sham. [file Image_2.tif]

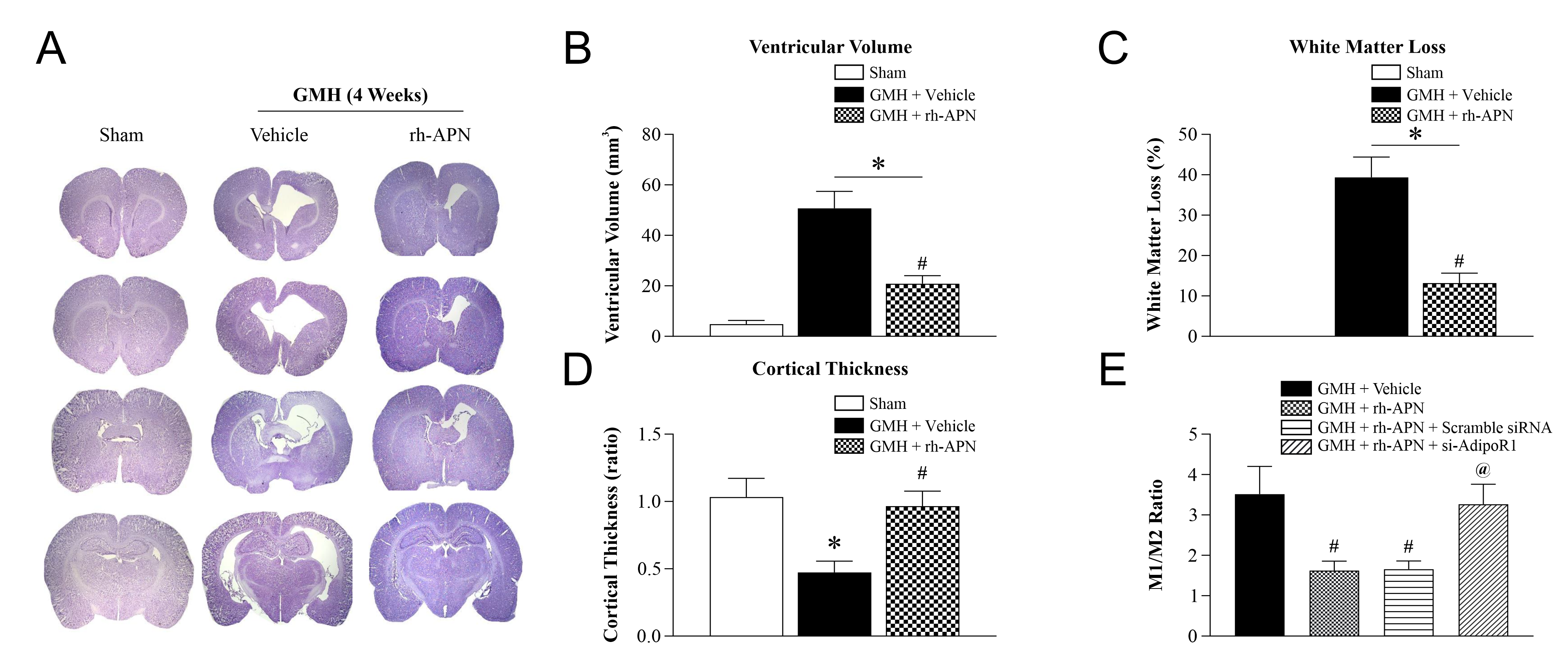

Supplement: Supplementary Figure 3 — (A–D) Quantifications of Nissl-stained brain sections showing rh-APN significantly reduced ventricular volume (A, B) and the white matter loss (C), while increased (D) cortical thickness in pups with GMH. (E) The ratio of CD68+Iba1+ M1-like cells and CD206+Iba1+ M2-like cells after GMH with rh-APN treatment or rh-APN + Scramble siRNA or rh-APN + si- AdipoR1. Values are expressed as mean ± SD. ANOVA, Dunnett. n = 8 for each group. *P < 0.05 compared to sham, #P < 0.05 compared to GMH + Vehicle, @P < 0.05 compared to GMH + rh-APN or GMH + rh-APN + Scramble siRNA. [file Image_3.tif]

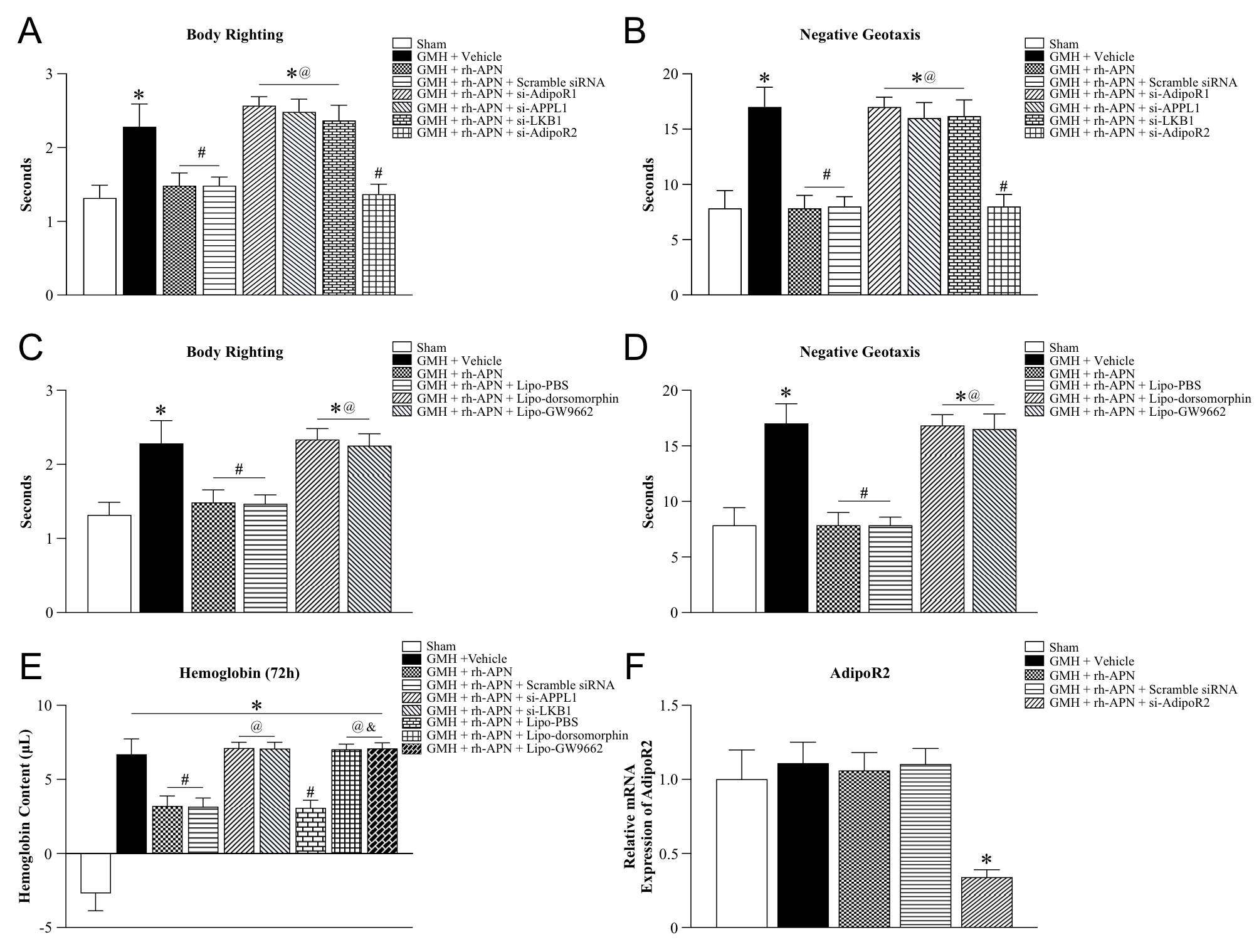

Supplement: Supplementary Figure 4 — (A) Righting reflex and (B) Geotaxis reflex tests showed that intracerebroventricular administration of si-AdipoR1, si-APPL1 and si-LKB1, but not si-AdipoR2, significantly reversed the protective effect of rh-APN when compared with their negative control (Scramble siRNA) group at day 3 after GMH. *P < 0.05 compared to sham, #P < 0.05 compared to GMH + Vehicle, @P < 0.05 compared to GMH + rh-APN or GMH + rh-APN + Scramble siRNA. (C) Righting reflex and (D) Geotaxis reflex tests showed that Lipo-dorsomorphin and Lipo-GW9662 significantly abolished the protective effect of rh-APN when compared with GMH + rh-APN + Lipo-PBS group at day 3 after GMH. *P < 0.05 compared to sham, #P < 0.05 compared to GMH + Vehicle, @P < 0.05 compared to GMH + rh-APN or GMH + rh-APN + Lipo-PBS. (E) Hemoglobin assays were conducted after intracerebroventricular administration of si-APPL1, si-LKB1, Lipo-dorsomorphin, Lipo-GW9662, and their negative control with treatment of rh-APN at 72 hours after GMH. *P < 0.05 compared to sham, #P < 0.05 compared to GMH + Vehicle, @P < 0.05 compared to GMH + rh-APN or GMH + rh-APN + Scramble siRNA. &P < 0.05 compared to GMH + rh-APN or GMH + rh-APN + Lipo-PBS. (F) The mRNA expression of AdipoR2 with the treatment of rh-APN, rh-APN + si-AdipoR2, or their negative control after GMH by qRT-PCR. *P < 0.05 compared to GMH + rh-APN or GMH + rh-APN + Scramble siRNA. [file Image_4.tif]
